# EVA-1 Functions as an UNC-40 Co-receptor to Enhance Attraction to the MADD-4 Guidance Cue in *Caenorhabditis elegans*

**DOI:** 10.1371/journal.pgen.1004521

**Published:** 2014-08-14

**Authors:** Kevin Ka Ming Chan, Ashwin Seetharaman, Rachel Bagg, Guillermo Selman, Yuqian Zhang, Joowan Kim, Peter J. Roy

**Affiliations:** 1 Department of Molecular Genetics, University of Toronto, Toronto, Ontario, Canada; 2 The Donnelly Centre for Cellular and Biomolecular Research, University of Toronto, Toronto, Ontario, Canada; 3 The Collaborative Programme in Developmental Biology, University of Toronto, Toronto, Ontario, Canada; University of California San Diego, United States of America

## Abstract

We recently discovered a secreted and diffusible midline cue called MADD-4 (an ADAMTSL) that guides migrations along the dorsoventral axis of the nematode *Caenorhabditis elegans*. We showed that the transmembrane receptor, UNC-40 (DCC), whose canonical ligand is the UNC-6 (netrin) guidance cue, is required for extension towards MADD-4. Here, we demonstrate that MADD-4 interacts with an EVA-1/UNC-40 co-receptor complex to attract cell extensions. EVA-1 is a conserved transmembrane protein with predicted galactose-binding lectin domains. EVA-1 functions in the same pathway as MADD-4, physically interacts with both MADD-4 and UNC-40, and enhances UNC-40's sensitivity to the MADD-4 cue. This enhancement is especially important in the presence of UNC-6. In EVA-1's absence, UNC-6 interferes with UNC-40's responsiveness to MADD-4; in UNC-6's absence, UNC-40's responsiveness to MADD-4 is less dependent on EVA-1. By enabling UNC-40 to respond to MADD-4 in the presence of UNC-6, EVA-1 may increase the precision by which UNC-40-directed processes can reach their MADD-4-expressing targets within a field of the UNC-6 guidance cue.

## Author Summary

During animal development, cells and cell extensions migrate along stereotypical paths to their target destination by interacting with guidance cues in their environment. The guidance receptors on the surface of these cells can each interact with several different cues, and many of the cues can each interact with multiple receptors. How a migrating cell can reach its target amid this apparent receptor-cue promiscuity is poorly understood. Here, we extend our earlier investigation of how the UNC-40 receptor, which is known to interact with the UNC-6 guidance cue, mediates attraction towards the MADD-4 guidance cue. We show that another transmembrane protein called EVA-1 increases the sensitivity of UNC-40 to MADD-4. This increase in sensitivity allows UNC-40 to respond to MADD-4 in the presence of UNC-6. Without EVA-1, UNC-6 dominates UNC-40 function and restricts its response to MADD-4. Hence, the presence of EVA-1 acts like a switch to change UNC-40's sensitivity from UNC-6 to MADD-4, and in turn allows UNC-40-expressing cells to migrate towards the source of MADD-4 within a field of the UNC-6 guidance cue.

## Introduction

A migrating cell or growth cone will likely encounter many different spatial cues as it advances towards its destination. For example, motor axons in the developing spinal cord encounter more than six different guidance cues as they exit the spinal column [1]. The response of the extending axons or migrating cells depends upon the complement of receptors that they express. However, several receptors are known to be capable of interacting with multiple cues, and many cues are known to bind multiple receptors [2], [3]. How migrating cells or cell extensions restrict their responses to distinct cues to generate stereotypical trajectories amidst this apparent receptor-ligand promiscuity is not well understood. Tackling this question with the nematode animal model *Caenorhabditis elegans* simplifies this problem because of its facile genetics and a limited number of paralogous guidance cues and receptors.

Our group uses specialized membrane projections from the body wall muscles of *C. elegans*, called muscle arms, as a model to better understand guided migration [4]. Ventral body wall muscles extend muscle arms to the ventral nerve cords and dorsal body wall muscles extend arms to the dorsal nerve cords [5]. Each muscle extends a stereotypical number of muscle arms that are guided to the nearest nerve cord [4]. Proper muscle arm extension is important because the muscle arm termini harbor the post-synaptic elements of the neuromuscular junction [5].

Muscle arm extension has proven to be a useful model of guidance because its study has led to the discovery of previously uncharacterized components that also play less obvious roles in axon guidance. We recently discovered that MADD-4, which is the sole *C. elegans* ortholog of the poorly understood non-enzymatic ADAMTSL superfamily of proteins, is the diffusible cue that is secreted by motor neurons to attract extending larval muscle arms [6]. The UNC-40 receptor (called DCC in vertebrates) and its adaptor protein MADD-2 (C1-family TRIM protein), are both required in muscles to direct muscle arm extension towards sources of MADD-4 [6], [7], [8]. UNC-40 is well known for its roles in directing migrations in response to its canonical ligand, UNC-6 (netrin), which is secreted by ventral cells during early development and is thought to form a gradient with a maximum at the ventral midline [9]. A role for UNC-6 in guiding ventral muscle arms is revealed only upon removal of MADD-4, suggesting that both ligands might function through UNC-40 to attract ventral muscle arms [6].

In addition to guiding muscle arms, MADD-4 and MADD-2 also play roles in guiding the AVM mechanosensory axon. The AVM cell body is located near the lateral midline and extends a single pioneer axon that is guided to the ventral midline by two pathways that function in parallel [10]. In one pathway, UNC-40 mediates AVM axon extension towards UNC-6 [11], [12]. In a parallel pathway, SAX-3 (robo) and its co-receptor EVA-1 mediate AVM axon repulsion from SLT-1 (slit), which is expressed by the dorsal muscles and is thought to form a gradient with a maximum at the dorsal midline [13], [14], [15]. EVA-1 is a conserved single pass transmembrane protein with a short cytoplasmic tail and an extracellular portion that encodes two predicted lectin-like galactose-binding domains [14]. We and others have demonstrated that MADD-4 and MADD-2 function in the UNC-40 pathway to ensure the AVM axon reaches its ventral midline target [6], [8], [16], [18].

Previous work has established that UNC-40's attractive response to the UNC-6 cue can be manipulated in at least two ways to achieve different outcomes. First, the UNC-5 receptor can subvert UNC-40's attractive response to UNC-6 to repulsion [18]. Second, SAX-3 may dampen UNC-40's sensitivity to UNC-6 by what is known as a set-point mechanism [14]. As SAX-3-expressing cells move away from the dorsal maxima of the SLT-1 ligand, SAX-3 is thought to become increasingly free from SLT-1 to antagonize UNC-40 activity. SAX-3 antagonism of UNC-40 activity may prevent signal saturation of the netrin pathway as the migrating process encounters increasing concentrations of UNC-6 as it approaches the ventral midline [14].

Here, we report a third mechanism by which UNC-40's activity is modulated. We found that EVA-1 is part of an UNC-40 co-receptor complex that functions to sensitize UNC-40 to the MADD-4 cue. By doing so, EVA-1 counteracts UNC-6's interference of UNC-40-mediated attraction towards MADD-4-expressing targets. This mechanism may enhance the ability of cell extensions to reach MADD-4-expressing targets within a field of the more globally distributed UNC-6 guidance cue.

## Results

### EVA-1 Functions in a MADD-4 Pathway

**Figure 1:**
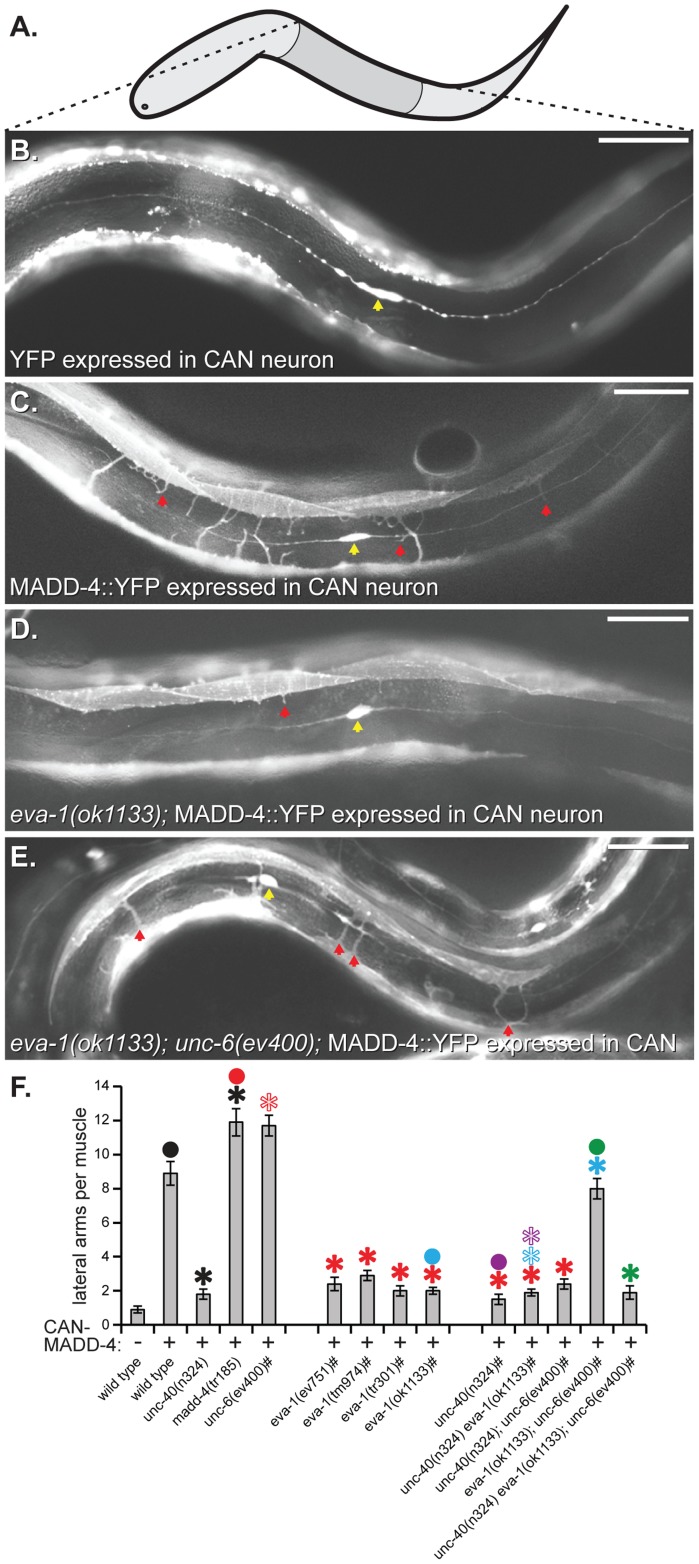
EVA-1 functions with UNC-40 to mediate muscle arm attraction to MADD-4. **A**. A schematic of the worm indicating the area shown in B-E. Anterior is to the left and dorsal is up. **B–E**. Lateral views of worms expressing a muscle membrane marker (*trIs30*) along with the indicated transgenic protein from the CAN neuron (yellow arrowhead). Examples of muscle arms extending into the lateral space are indicated with a red arrowhead. Anterior is to the left and dorsal is up. **F**. Quantification of muscle arm extension into the lateral space in response to CAN-expressed MADD-4::YFP (CAN-MADD-4). The pound sign (#) next to the x-axis labels indicate that these animals are also mutant for *madd-4(tr185)*, which increases the sensitivity of the assay. All of the alleles used are predicted to be null for the indicated gene, except for *tr185*, which is a null allele of the *madd-4b* isoform. Statistical significance (*p*<0.01) is indicated with a solid asterisk that is matched in colour with a dot above the data point to which the comparison was made. An outlined asterisk indicates a lack of significance (*p*>0.01). CAN expression is driven by the *ceh-23* promoter for all transgenes presented here. The scale bar shows 50 micrometers. Standard error of the mean is shown in all graphs.

To identify new components that facilitate MADD-4 attraction, we performed a forward genetic screen for mutants that disrupt the ability of ectopic MADD-4 to attract muscle arms. We used a previously described strain [6] that expresses MADD-4 from a pair of bilaterally-symmetrical CAN neurons (Figure 1a–1c). In this strain, muscle arms are redirected towards the lateral source of MADD-4 in an UNC-40-dependent fashion [6]. We also incorporated a *madd-4* null mutation in the screening strain as it increases the number of processes directed towards laterally-expressed ectopic MADD-4 and therefore creates a sensitized background (see Figure 1f for example). We screened approximately 22,000 randomly mutagenized genomes and identified 44 penetrant mutants that suppressed redirected muscle arms without obviously diminishing the expression of the transgenes from the CAN neuron. We isolated 9 mutants that are likely new alleles of *unc-40* or *unc-73* based on their characteristic uncoordinated phenotype and commissural axon guidance defects, and 15 mutants with mutations in *madd-2* (not shown). Given our previous characterization of these gene classes [7], [8], we did not pursue these mutants further. Among the remaining 20 mutants, one represented a novel allele of *eva-1* that we call *tr301. tr301* mutates *eva-1's* Q86 codon to non-sense. We subsequently found that all known *eva-1* alleles are suppressors of muscle arm extension towards ectopic MADD-4 (Figures 1d and 1f). All of the primary and raw data that is displayed in our figures can be found in Dataset S1 and Dataset S2, respectively.

**Figure 2:**
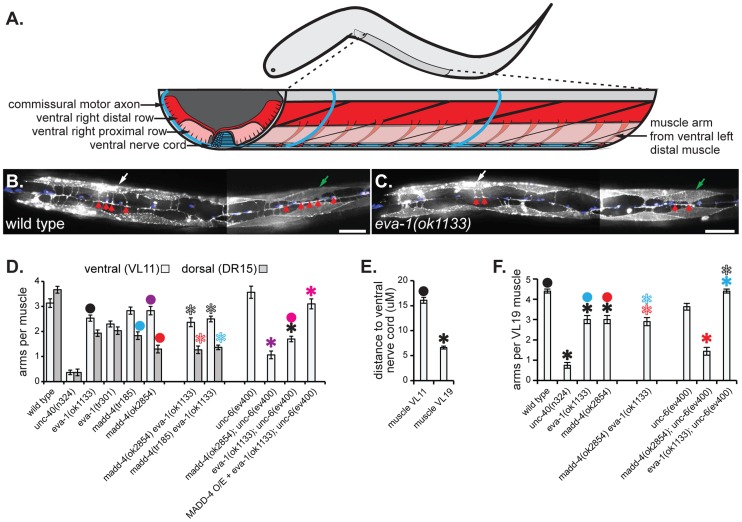
EVA-1 functions in a MADD-4 pathway to counteract UNC-6 interference. **A**. An illustration of the worm that schematizes the muscle arms of ventral left distal body wall muscles. Anterior is to the left and dorsal is up. **B & C**. Micrographs showing a ventral view of a young adult worm of the indicated genotype with anterior to the left. The yellow arrowhead indicates the ventral cord, the white and green arrows indicates ventral left muscle numbers 11 and 19 (VL11 and VL19), respectively. The red arrowheads indicate the muscle arms of VL11 and VL19. The scale bar shows 50 µM. **D**. Quantification of muscle arm extension from VL11 and dorsal right muscle number 15 (DR15) in young adults of the indicated genotype. Dorsal muscle arm data is not shown for strains carrying the *unc-6* mutation because they lack motor neurons within the dorsal cord, which consequently confounds any interpretation of the resulting muscle arm phenotypes. In the last column, MADD-4 is over-expressed (O/E) pan-neuronally from the *trIs66* integrated transgenic array (see the materials and methods section for details). **E**. The average distance of the mid-proximal face of the indicated muscle to the ventral nerve cord in wild type animals (that also harbor the *trIs30* muscle arm and neuronal markers). **F**. Quantification of VL19 muscle arm extension in young adults of the indicated genotype. Statistical significance (*p*<0.001) is indicated with a solid asterisk which is matched with a dot above the data point to which the comparison was made. Standard error of the mean is shown in all graphs.

We examined the role of *eva-1* in normal muscle arm extension towards the midline motor neurons and found a significant reduction compared to wild type (Figure 2a–2c). *eva-1* mutants have more dorsal muscle arm defects than ventral muscle arm defects, which is similar to *madd-4* mutants [6]. We therefore used double mutant analysis to ask whether EVA-1's role in muscle arm extension might be restricted to the MADD-4 pathway. *madd-4 eva-1* double mutants have muscle arm extension defects that are no more severe than the strongest defect of either single mutant alone (Figure 2d). Other allelic combinations were found to yield similar results (not shown). Given that muscle arm extension defects can be more severe than that observed in the *madd-4 eva-1* double mutant, this result indicates that EVA-1 and MADD-4 function in the same pathway to guide muscle arms.

To further explore the genetic relationship between *eva-1* and *madd-4*, we constructed additional double mutants with other genes known to regulate muscle arm extension. The near absence of muscle arm extension towards the midline in the *unc-40* null mutant precludes its use in double mutant analyses to reliably determine whether a second gene product likely functions with UNC-40. However, we previously demonstrated that mutations in *unc-6*, which do not elicit ventral muscle arm defects on their own, enhance the ventral muscle arm extension defects of the *madd-4* null mutant, suggesting that UNC-6 plays a role in attracting muscle arms to the ventral midline in MADD-4's absence [6] (Figure 2d). We found that the *unc-6* null mutant also enhances the ventral muscle arm defects of *eva-1* (Figure 2d), consistent with the idea that MADD-4 and EVA-1 function together in a pathway that operates in parallel to UNC-6. Of note, we do not examine dorsal muscle arm extension in the *unc-6* null mutant backgrounds because these animals lack motor neurons within the dorsal cord, which consequently confounds the interpretation of resulting muscle arm phenotypes.

### EVA-1 Is a MADD-4 Receptor

**Figure 3:**
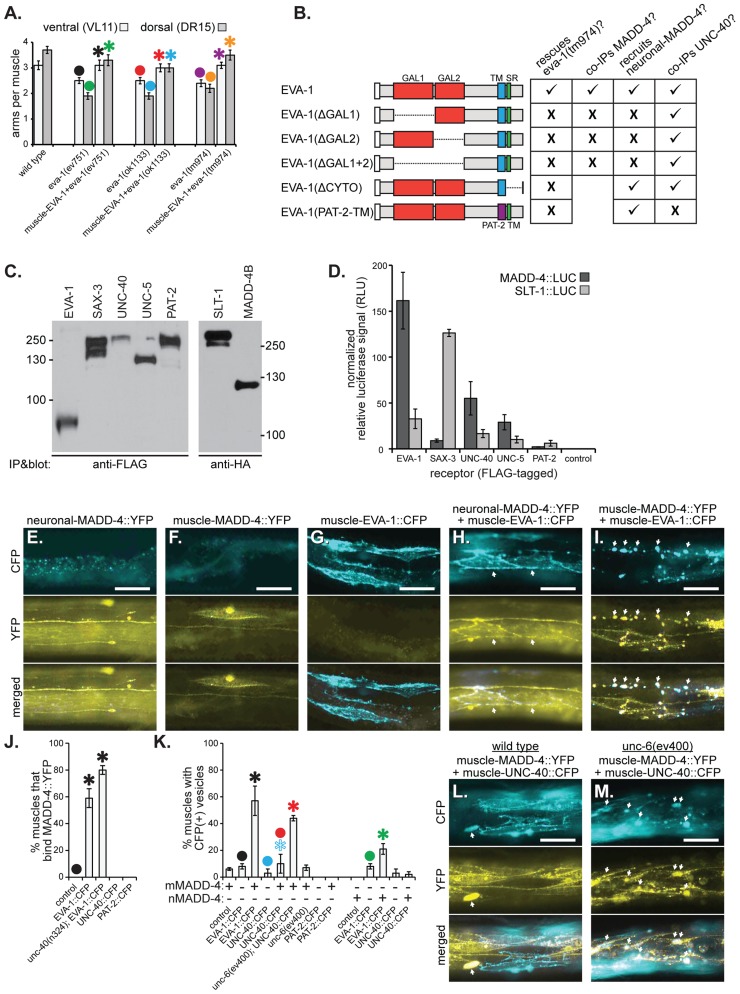
EVA-1 functions cell-autonomously in muscles and interacts with MADD-4. **A**. Muscle-expressed EVA-1::CFP rescues the muscle extension defects of *eva-1* mutants. **B**. A summary of EVA-1 domain function that is fully detailed in Figure S1. **C&D**. FLAG-tagged receptors were expressed from HEK293 cells, bathed in conditioned media from other HEK293 cells that express HA- and Gaussia luciferase-tagged MADD-4 or SLT-1 ligands, and immunoprecipitated to determine the relative amounts of ligand that co-immunoprecipitates with the receptor (see the materials and methods section for more details). **C**. The western blot on the left shows the five immunoprecipitated FLAG-tagged receptors. The western blot on the right shows the two HA- and Gaussia luciferase-tagged ligands that were collected from cell culture. **D**. The normalized relative levels of luciferase signal that immunoprecipitated with each potential ligand-receptor complex. **E–I**. Shown are animals harbouring one of three different transgenes that drive the expression of either neuronally-expressed MADD-4::YFP (from the *trIs66* transgenic array) (**E**), muscle-expressed MADD-4::YFP (from the *trIs78* transgenic array) (**F**), or muscle-expressed EVA-1::CFP (from the *trIs89* transgenic array) (**G**), or animals harbouring two of the transgenes; *trIs66* and *trIs89* (**H**) and *trIs78* and *trIs89* (**I**). The relative levels of MADD-4::YFP expression from *trIs66* and *trIs78* is shown in Figure S2a. Images show either the CFP channel (top), YFP channel (middle) or a merged view (bottom). Arrows in ‘H’ indicate the localization of MADD-4::YFP to EVA-1::CFP expressing muscles; arrows in ‘I’ indicate the vesicularization of MADD-4::YFP and EVA-1::CFP in the muscle cells. **J**. The quantification of neuronally-secreted MADD-4::YFP localization to muscles over-expressing the indicated receptor. **K**. The quantification of CFP vesicles in animals that over-express the indicated CFP-tagged receptors (x-axis) in muscles in either the presence of MADD-4::YFP expressed from dorsal muscles (mMADD-4) or pan-neuronally (nMADD-4). The colocalization of MADD-4 and EVA-1 with the RAB-11 and RAB-5 endosomal markers are shown in Figure S2b and S2c. **L&M**. MADD-4::YFP fails to induce obvious vesicularization of UNC-40::CFP in a wild type background (L), but YFP-CFP vesicles are obvious in animals that lack UNC-6 (M). In A, J, and K, statistical significance (*p*<0.05) is indicated with a solid asterisk which is matched with a dot above the data point to which the comparison was made. In all micrographs, the scale bar represents 50 micrometers. In all graphs, standard error of the mean is shown.

Previous work showed that *eva-1* is likely expressed in several tissues, including muscle [14]. We tested whether *eva-1* functions cell-autonomously by expressing an EVA-1::CFP fusion protein specifically in the body wall muscles and found that it could rescue the muscle arm defects of *eva-1* mutants (Figure 3a). These data indicate that, like UNC-40 [7], EVA-1 functions cell-autonomously to direct muscle arm extension. A systematic analysis of the functional requirement of EVA-1's domains indicate that each of its two predicted galactose-binding lectin-like domains, its transmembrane domain, and its cytoplasmic tail are required for muscle arm extension (Figures 3b and S1a).

Given that: i) EVA-1 is a single-pass transmembrane protein that functions in muscle to direct muscle arm extension, and that ii) our genetic analyses indicate that MADD-4 and EVA-1 function in the same pathway, we hypothesized that EVA-1 may be a receptor for MADD-4. We therefore investigated whether MADD-4 might interact physically with EVA-1. We expressed luciferase-tagged MADD-4 and SLT-1 (as a control) from HEK293 cells and collected the conditioned medium (Figure 3c). We overlaid this conditioned medium onto cells expressing FLAG-tagged EVA-1, UNC-40, SAX-3, UNC-5, PAT-2 (α integrin), or control cells not expressing any *C. elegans* proteins (Figure 3c). We then immunoprecipitated the receptors and measured luciferase activity in the washed immunoprecipitate. We found that MADD-4 had obvious affinity for EVA-1-expressing cells, some affinity for UNC-40 and UNC-5-expressing cells, and negligible affinity for SAX-3 and PAT-2-expressing cells (Figure 3d). By contrast, SLT-1 had obvious affinity for SAX-3-expressing cells, some affinity for EVA-1-expressing cells, and negligible affinity for UNC-40, UNC-5, and PAT-2-expressing cells (Figure 3d). Consistent with the functional necessity of both of EVA-1's galactose-binding lectin-like domains, EVA-1 requires both of these domains to be able to co-immunoprecipitate MADD-4 (Figures 3b and S1b).

We next analyzed MADD-4 interactions with EVA-1 and UNC-40 *in vivo*. When expressed from the nervous system, MADD-4::YFP localization appears restricted to neurons (Figure 3e). However, when expressed from the nervous system of animals that also express EVA-1::CFP in muscles, MADD-4::YFP localizes to the EVA-1::CFP-expressing muscles (Figures 3e–3h and 3j). The ability of muscle-expressed EVA-1::CFP to relocalize neuronally-secreted MADD-4::YFP to muscles is independent of UNC-40 (Figure 3j). Consistent with our co-immunoprecipitation analyses, both of EVA-1's galactose-binding lectin-like domains are required for muscle-expressed EVA-1 to recruit neuronal-expressed MADD-4 (Figures 3b and S1c). We observe negligible recruitment of neuronally-expressed MADD-4::YFP to muscles over-expressing UNC-40 (Figure 3j).

In animals expressing MADD-4::YFP from neurons and EVA-1::CFP in muscles, we observe the coincidental vesicularization of YFP and CFP (Figure 3k). When MADD-4::YFP is expressed from the same EVA-1::CFP-expressing muscles, more MADD-4::YFP/EVA-1::CFP vesicles are apparent (Figures 3i and 3k). The increase in vesicles when MADD-4::YFP is expressed from muscles is coincidental with a higher level of expression of MADD-4::YFP in this strain (Figure S2a) and increased proximity to the EVA-1::CFP expressed on muscles. An early endosomal marker (RAB-5) and a recycling endosomal marker (RAB-11) [19] coincide with the MADD-4::YFP/EVA-1::CFP-containing vesicles (Figures S2b and S2c), suggesting that the MADD-4-EVA-1 complex is internalized. These results provide further support for the idea that MADD-4 interacts with EVA-1 *in vivo*. We observe negligible vesicularization of MADD-4::YFP with UNC-40::CFP (Figures 3k and 3l). Together, our genetic, transgenic, cell biological, and co-immunoprecipitation analyses support a model whereby EVA-1 acts as a receptor for MADD-4.

### EVA-1 Functions within an UNC-40 Complex

**Figure 4:**
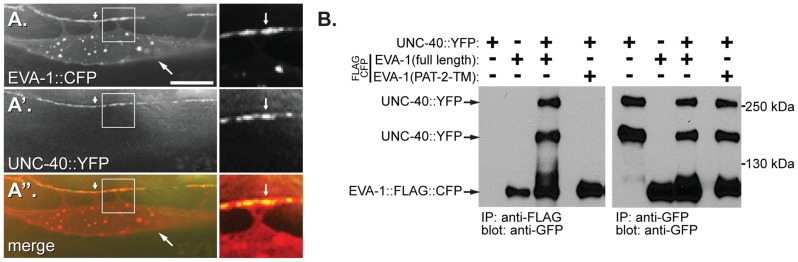
EVA-1 interacts with UNC-40 via its transmembrane domain. **A, A′** and **A″**. Shown is a single muscle cell that expresses EVA-1::CFP (expressed from an extra-chromosomal array) (**A**) that is enriched at muscle arm termini (arrowhead), and functional UNC-40::YFP (expressed from the *trIs34* array) (**A′**), and their co-localization (**A″**). The white arrow in the left-hand panels points to an example of muscle arm termini. The area that is boxed in each left-hand panel is respectively magnified in each right-hand panel. The arrow in the right-hand panels indicates one area of co-localization. The lack of dependence of EVA-1 and UNC-40 localization on the presence of UNC-40 and EVA-1, respectively, is shown in Figure S3a and S3b. The scale bar represents 50 µM. **B**. A western blot showing that UNC-40 co-immunoprecipitates with full-length FLAG-tagged EVA-1, but not with a version of EVA-1 whose transmembrane domain has been swapped for that of PAT-2. Control blots showing a lack of interaction between EVA-1 and PAT-2 and between UNC-40 and PAT-2 are shown in Figure S3c and S3d.

EVA-1 and UNC-40 are both required for muscle arm extension towards MADD-4 (Figure 1f). We therefore hypothesized that the two transmembrane proteins might function in a co-receptor complex to mediate MADD-4 activity. To investigate this possibility, we first examined whether transgenic EVA-1 and UNC-40 co-localize with one another in muscle cells and found that they do (Figure 4a). Of note, the absence of EVA-1 does not affect the subcellular localization UNC-40 and vice versa (Figure S3a and S3b).

Next, we investigated whether EVA-1 and UNC-40 could be found in the same complex. We expressed functional epitope-tagged EVA-1 and UNC-40 in *C. elegans* muscles and tested whether they co-immunoprecipitate and found that they do (Figure 4b). By contrast, neither UNC-40 nor EVA-1 co-immunoprecipitates with PAT-2, a negative control (Figures S3c and S3d). A systematic analysis of EVA-1 domains revealed that only the alteration of EVA-1's transmembrane domain disrupts its ability to co-immunoprecipitate UNC-40; swapping EVA-1's transmembrane domain for that of PAT-2's abolishes the co-immunoprecipitation of EVA-1 and UNC-40 (Figures 4b and 3b). The version of EVA-1 with its transmembrane domain swapped for that of PAT-2 cannot rescue the muscle arm defects of the *eva-1* null mutant (Figure 3b and S1a), despite being subcellularly localized to the plasma membrane of muscles, and being able to recruit neuronally-secreted MADD-4::YFP to muscles (Figures 3b and S1c). This suggests that the interaction between EVA-1 and UNC-40 is functionally relevant. Together, these results support the idea that EVA-1 functions within an UNC-40 complex.

### UNC-6 Interferes with UNC-40's Interaction with MADD-4

**Figure 5:**
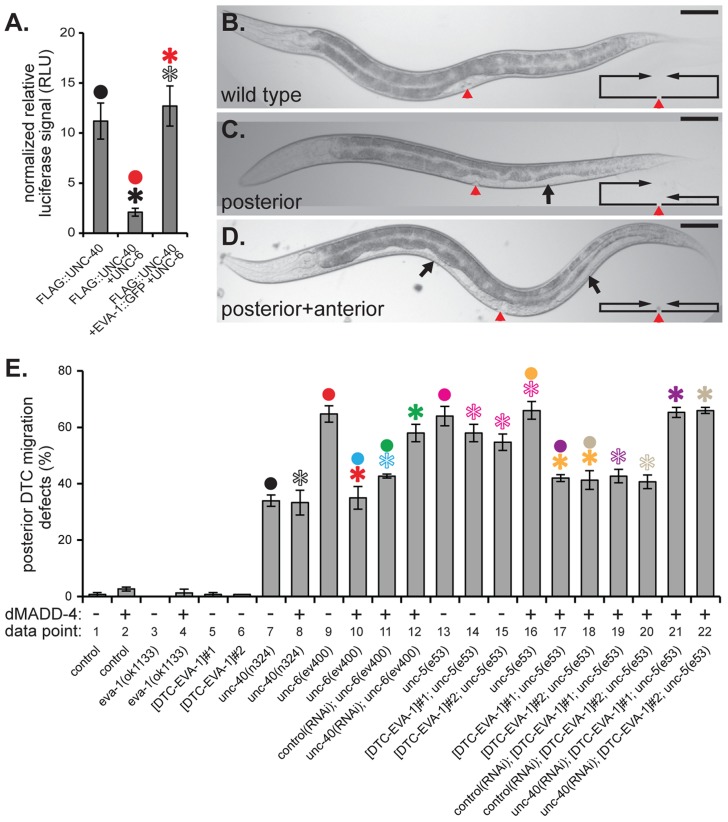
EVA-1 is sufficient to confer sensitivity of UNC-40 to MADD-4 in the background of UNC-6. **A**. Co-expression of UNC-6 interferes with the ability of HEK293-expressed FLAG-tagged UNC-40 to co-immunoprecipitate luciferase-tagged MADD-4. Co-expression of EVA-1 relieves UNC-6's interference of the MADD-4-UNC-40 interaction. **B–D**. Examples of the phenotypic consequence of wild-type (B) and errant distal tip cell migration (C&D). In the micrographs, the red arrowhead indicates the vulval slit, and the black arrows indicate the white ventral patch that results from a guidance error in distal tip cell migration. The line/arrow schematics in the lower right of each micrograph chart the migratory path of the anterior and posterior distal tip cells in the respective animal. Dorsal is up and anterior is to the left. In all micrographs, the scale bar represents 50 µM. **E**. Distal tip cell migration defects either in the presence (+) or absence (−) of dorsal muscle-expressed (m)MADD-4 (from the *trIs78* transgenic array) for the indicated genotype. For simplicity, only the posterior distal defects are shown; the anterior defects are reported in supplemental Figure S4 and show similar trends. The purpose of the ‘data point’ row is to provide clarity within main text. EVA-1::CFP was expressed in the distal tip cells from the *emb-9* promoter as was previously done for UNC-5 [28]. The two extrachromosomal arrays are called *trEx948#1* ([DTC-EVA-1]#1 on the graph) and *trEx948#2* ([DTC-EVA-1]#2 on the graph). For each graph, statistical significance (*p*<0.01) is documented as described for figure 1f. Standard error of the mean is shown in both graphs.

UNC-40 localizes to the tips of muscle arms [7], is required for muscle arm extension towards normal and ectopic sources of MADD-4 (Figure 1f) [6], and can co-immunoprecipitate MADD-4 when expressed from HEK293 cells (Figure 3d). However, we did not observe an obvious *in vivo* association between MADD-4::YFP and muscle-expressed UNC-40::CFP in worms (Figures 3j–3l). Given that UNC-6 is UNC-40's canonical ligand, we tested the idea that MADD-4's ability to interact with UNC-40 is restricted by UNC-6. Upon genetic removal of UNC-6, we observed an obvious increase in the vesicularization of muscle-expressed UNC-40::CFP by MADD-4::YFP (Figures 3k and 3m), suggesting that MADD-4 and UNC-40 are capable of interacting *in vivo*. We also tested whether UNC-6 also interferes with MADD-4's interaction with HEK293-expressed UNC-40 *in culture* and found that it did (Figure 5a). Together, these results suggest that UNC-40 can serve as a MADD-4 receptor and that UNC-6 can interfere with the MADD-4-UNC-40 interaction.

### EVA-1 Sensitizes UNC-40 to the MADD-4 Cue

Above, we demonstrated that: i) EVA-1 and UNC-40 are needed for muscle arm extension towards endogenous and ectopic sources of MADD-4; ii) EVA-1 likely functions within an UNC-40 co-receptor complex; iii) In the absence of MADD-4, UNC-6 has demonstrable activity in attracting muscle arms to the ventral nerve cord; and iv) UNC-6 is capable of interfering with the MADD-4-UNC-40 interaction. Together, these observations led us to hypothesize that EVA-1's role might be to counteract the UNC-6 interference by sensitizing UNC-40 to the MADD-4 cue. We tested this hypothesis in several ways.

First, we returned to our assay in which we redirect arms laterally by ectopically-expressing MADD-4 from the CAN neuron (Figure 1). If EVA-1's role in mediating MADD-4 attraction is to counteract UNC-6 interference, then the reason why EVA-1's absence suppresses muscle arm extension towards CAN-expressed MADD-4 is because ventral sources of UNC-6 are interfering with UNC-40-mediated attraction to the CAN-expressed MADD-4. If true, then removal of UNC-6 should restore muscle arm extension towards CAN-expressed MADD-4 in the *eva-1* null mutant background. Indeed, we observed restoration of lateral muscle arm extension towards ectopic MADD-4 in the *eva-1; unc-6* double null mutants (Figures 1e and 1f). Lateral muscle arm extension remains dependent on UNC-40 in the *eva-1; unc-6* double mutants (Figure 1f). These observations support the model whereby EVA-1 sensitizes UNC-40 to MADD-4 and this sensitization is necessary to counteract UNC-6's interference.

Next, we investigated whether our model holds true for muscle arms that extend towards the endogenous source of MADD-4 at the ventral nerve cord. We already knew that genetic removal of UNC-6 does not restore muscle arm extension towards the ventral nerve cord in *eva-1* null mutants and instead enhances the defect (Figure 2d). Although this provides evidence that UNC-6 and EVA-1 function in parallel to guide arms to the ventral cord, this observation is seemingly incongruent with the idea that UNC-6 interferes with the MADD-4 UNC-40 interaction. However, we reasoned that endogenous levels of MADD-4 from the ventral nerve cord might not be high enough to engage UNC-40 without EVA-1 present on the body wall muscle cell that we normally study, which is called ventral left muscle number 11 (VL11) ([4] and Figure 2b). Indeed, when more MADD-4 is expressed from the nervous system (from the *trIs66* transgenic array), the VL11 muscle arm defects of the *eva-1 unc-6* double null mutant are rescued (Figure 2d). In addition, we examined muscle arm extension from a muscle (VL19) that is located ∼10 micrometers closer to the ventral nerve cord than VL11 (Figure 2e) and is consequently likely to encounter more endogenous MADD-4. We found that the pattern of genetic interactions exhibited by VL19 is the same as the pattern that we uncovered in the ectopic assay; the genetic removal of UNC-6 suppresses the muscle arm defects of the *eva-1* null mutant (Figure 2f). Hence, EVA-1 counteracts UNC-6's interference of UNC-40-mediated guidance towards MADD-4 from either ectopic or endogenous sources.

Notably, in animals that lack UNC-6, endogenous MADD-4 is sufficient to attract muscle arms from the muscle that is closer to it (VL19), but requires EVA-1 to attract muscle arms from the muscle that is further from it (VL11) (Figures 2d–2f). This suggests that EVA-1's ability to sensitize UNC-40 to MADD-4 is independent of UNC-6.

### EVA-1 Is Sufficient to Sensitize UNC-40 to MADD-4 in the Background of UNC-6

Above, we demonstrated that EVA-1 is necessary to facilitate UNC-40 sensitivity to MADD-4, and that UNC-40's enhanced sensitivity to MADD-4 is especially important in the presence of UNC-6. We took two approaches to determine whether EVA-1 is also sufficient for this activity. First, we tested whether EVA-1 can counteract UNC-6's interference of the MADD-4-UNC-40 interaction that we observed in cell culture. Upon co-expressing EVA-1 with UNC-40 in HEK293 cells, we are indeed able to counteract UNC-6's interference of UNC-40's interaction with MADD-4 (Figure 5a).

Next, we examined EVA-1's ability to modulate an UNC-40-dependent cell migration that normally doesn't require EVA-1. The expansion of the bi-lobed somatic gonad during *C. elegans* development is led by the migratory distal tip cell at the leading edge of each gonad lobe [20]. The distal tip cell leads a ventral to dorsal gonad expansion via the expression of UNC-40 and UNC-5 co-receptors that mediate a repulsive migration away from the ventral source of UNC-6 (Figure 5b). Genetic removal of any of these components results in a gonad that often fails to reach the dorsal body wall and instead expands ventrally, which in turn, results in a white patch on the animal's ventral side (Figures 5b–5d). EVA-1 is not obviously expressed in the distal tip cells [14], and loss of EVA-1 has no consequence on the migration of the distal tip cell (Figures 5e and S4-data point 3), suggesting that EVA-1 plays no major role in distal tip cell migration. Given that the distal tip cells express UNC-40 [11], we tested whether dorsally-expressed MADD-4 could rescue the distal tip cell migration defect of the netrin pathway mutants. If our model of EVA-1/UNC-40/MADD-4/UNC-6 interactions are true, then dorsal expression of MADD-4 should only be able to rescue distal tip cell migration defects in the absence of UNC-6. Indeed, dorsally-expressed MADD-4 can significantly rescue the distal tip cell migration defects of animals that lack UNC-6 (Figures 5e and S4-data points 9 and 10), but not those that lack UNC-40 or UNC-5 (Figures 5e and S4- data points 7 and 8, and columns 13 and 16, respectively). However, expressing EVA-1 ectopically in the distal tip cell of *unc-5* mutants allows the migrating cell to respond to dorsally-expressed MADD-4 despite the presence of endogenous UNC-6 (Figures 5e and S4- data points 13–18). As expected, the ability of distal tip cell-expressed EVA-1 to confer sensitivity to the dorsally-expressed MADD-4 cue is dependent on UNC-40 (Figures 5e and S4- data points 19–22). Together, our results indicate that EVA-1 is both necessary and sufficient to sensitize UNC-40 to the MADD-4 cue. Furthermore, this enhanced sensitivity is necessary and sufficient to overcome UNC-6's interference of the UNC-40-mediated attraction towards MADD-4.

### EVA-1 and UNC-40 Direct Axon Extension towards MADD-4

**Figure 6:**
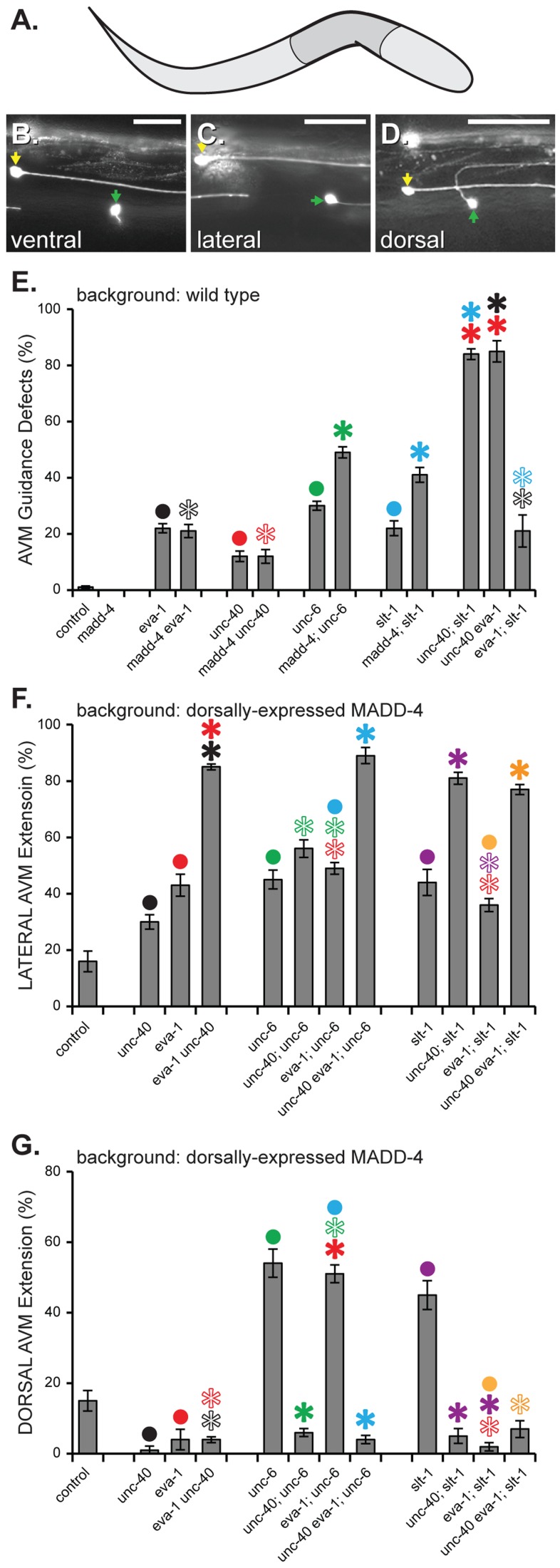
MADD-4 attracts extending AVM axons via EVA-1 and UNC-40. **A**. A schematic illustrating the area of the worm shown in B-D. Anterior is to the right and dorsal is up. **B–D**. Three worms expressing MADD-4::YFP from the dorsal muscles (from the *trIs78* transgenic array) in which the AVM axon (green arrowhead) extends ventrally (B), laterally (C) or dorsally (D). The ALMR neuron (yellow arrowhead) is also seen in all three panels. The *muIs32* transgene is used to visualize the AVM and ALMR neurons [22]. The scale bar represents 50 µM. **E**. A genetic analysis of the AVM axon guidance errors in the indicated genetic background without transgenic expression of MADD-4. In all of these loss-of-function mutant backgrounds, the misguided axons invariably extend laterally. **F & G**. An analysis of laterally (F) or dorsally (G) -directed AVM axon extension in response to dorsally-expressed MADD-4::YFP. Shown is the percentage of animals in which the AVM extends laterally or dorsally, respectively. Note that in the background of dorsally-expressed MADD- 4, the AVM axon already extends dorsally in more than half of the *eva-1; unc-6* double mutant animals and leaves no room for the expected enhancement of *eva-1'*s lateral axon guidance defects by *unc-6*. Statistical significance is documented as described for figure 1f. The alleles used in this analysis are *madd-4(ok2854)*; *unc-40(n324), eva-1(ok1133), slt-1(ok255), and sax-3(ky200)*. Standard error of the mean is shown in all graphs.

We were curious to know whether EVA-1 might play a similar role in guiding axons towards MADD-4 as it does in guiding muscle arms. We previously demonstrated that a *madd-4* null mutation can enhance the AVM mechanosensory axon guidance defects of a *slt-1* or *unc-6* null mutant, but not those of an *unc-40* null mutant [6] (Figures 6a–6d). Here, we found that *madd-4* also failed to enhance the AVM guidance defects of an *eva-1* mutant (Figure 6e), suggesting that EVA-1 and UNC-40 may function together in a third pathway that is independent of SLT-1 and UNC-6 to mediate attraction towards MADD-4. Note that the AVM guidance defects reported in Figure 6e are invariably lateral extensions like that shown in Figure 6c.

We next examined the requirement for these factors in guiding the AVM axon towards heightened MADD-4 expression from the dorsal muscles, which attracts the AVM axon from its normal ventral trajectory to lateral and dorsal trajectories [6] (Figures 6c and 6d). Upon considering only the lateral guidance errors, the *eva-1* null is enhanced by the *unc-40* null, and not enhanced by the *slt-1* null (Figure 6f). These interactions are entirely consistent with a previous report that shows that EVA-1 can act as SAX-3's co-receptor for the SLT-1 ligand and that this pathway acts in parallel to one that includes UNC-40 and UNC-6 [14].

A distinct set of relationships between EVA-1, UNC-40, UNC-6 and SLT-1 become evident upon considering only the dorsal guidance errors of the AVM axon like that shown in Figure 6d. We previously found that *unc-40* is required for the dorsal redirection of the AVM axon towards dorsally-expressed MADD-4 [6]. We found that *eva-1* is also required for this dorsal redirection and that the *unc-40 eva-1* double mutant is as compromised in dorsal redirection of the AVM axon as the single *unc-40* mutant (Figure 6g). By contrast, eliminating the dorsal repulsive cue (SLT-1) or the ventral attractive cue (UNC-6) dramatically enhances AVM axon extension towards dorsally-expressed MADD-4 [6] (Figure 6g). These results are consistent with the idea that EVA-1 also functions with UNC-40 in a pathway that is distinct from SLT-1 and UNC-6 to mediate the extension of the AVM axon towards MADD-4.

Finally, we tested whether UNC-6 interferes with the UNC-40-mediated AVM axon extension towards MADD-4, and concurrently, whether EVA-1 counteracts UNC-6's interference. Indeed, we found that AVM axon guidance towards dorsally-expressed MADD-4 is suppressed in animals lacking EVA-1, but is restored in these *eva-1* null mutants upon removing UNC-6 (Figure 6g). We tested two other allelic combinations of *eva-1* and *unc-6* and found similar results (not shown). Genetic removal of UNC-40 in the background of the *eva-1; unc-6* double reduces AVM axon attraction to the levels seen in the *eva-1* single mutant, demonstrating that guidance towards MADD-4 remains dependent on UNC-40 in the absence of EVA-1 and UNC-6 (Figure 6g). As a control, we tested whether genetic removal of SLT-1 is able to restore AVM axon guidance towards dorsally-expressed MADD-4 in the *eva-1* mutant background and found that it cannot (Figure 6g). These results show that UNC-6 interferes with UNC-40's interaction with MADD-4 with not only in the context of muscle arm extension, but during axon guidance as well. In both cell types, EVA-1's ability to sensitize UNC-40 to MADD-4 is needed to overcome UNC-6's interference of the UNC-40-MADD-4 interaction.

## Discussion

Here, we provided evidence to support three main conclusions: Our first main conclusion is that the EVA-1 transmembrane protein interacts with the MADD-4 cue. We have provided numerous lines of evidence in support of this conclusion, including the following: i) EVA-1 functions cell-autonomously; ii) EVA-1 localizes to the plasma membrane of muscle arm extensions; iii) EVA-1 is necessary and sufficient to enable UNC-40-mediated attraction towards MADD-4 in the presence of UNC-6; iv) EVA-1 functions in the same genetic pathway as MADD-4; v) muscle-expressed EVA-1 is sufficient to recruit neuronally-expressed MADD-4; vi) EVA-1 co-immunoprecipitates MADD-4; and vii) EVA-1 interacts with MADD-4 via its two galactose binding lectin-like domains.

Our second main conclusion is that EVA-1 and UNC-40 function within a co-receptor complex to mediate attraction towards MADD-4. We also provided numerous lines of evidence in support of this second conclusion, including: i) Both EVA-1 and UNC-40 are necessary to mediate attraction towards MADD-4; ii) In the presence of UNC-6, neither EVA-1 nor UNC-40 alone is sufficient to mediate attraction towards MADD-4; iii) EVA-1 and UNC-40 likely function in the same pathway; iv) EVA-1 co-immunoprecipitates with UNC-40 and does so via its transmembrane domain; and v) EVA-1's transmembrane domain cannot be substituted with another transmembrane domain and preserve EVA-1 function, suggesting that EVA-1's interaction with UNC-40 via its transmembrane domain is functionally relevant.

Our third main conclusion is that EVA-1 sensitizes UNC-40 to the MADD-4 cue and that this heightened sensitivity is necessary to counteract UNC-6's interference of UNC-40-mediated attraction towards MADD-4. Again, we have presented numerous lines of evidence in support of this conclusion, including: i) EVA-1 is necessary to prevent UNC-6's interference of UNC-40-mediated muscle arm, axon, and distal tip cell extension towards endogenous and/or ectopic MADD-4; ii) EVA-1 is sufficient to enable UNC-40-mediated attraction towards MADD-4 in the presence of UNC-6 *in vivo*; and iii) EVA-1 can counteract UNC-6's interference of UNC-40's interaction with MADD-4 in HEK293 cell culture experiments.

Several lines of evidence suggest that EVA-1's role in transducing MADD-4 activity is entirely dependent on UNC-40. First, in the absence of UNC-40, muscle arm extension and axon extension towards MADD-4 is abolished, suggesting that EVA-1 cannot mediate attraction towards MADD-4 without UNC-40. Second, the suppression of muscle arm extension and axon guidance towards ectopic MADD-4 in animals that lack UNC-40 cannot be further suppressed by removing EVA-1. This suggests that EVA-1 and UNC-40 function together to mediate attraction towards MADD-4. Third, in normal circumstances, MADD-4 activity is dependent on both EVA-1 and UNC-40. Hence, if EVA-1 had an UNC-40-independent role in muscle arm extension, then genetic removal of EVA-1 would enhance the muscle arm defects of the *madd-4* null mutant. Because the *eva-1* null mutation fails to enhance the *madd-4* mutant, EVA-1 must not have a role in muscle arm extension that is independent of UNC-40. Fourth, in special circumstances (i.e. in animals without UNC-6), MADD-4 attraction can be mediated by UNC-40 in an EVA-1-independent manner. This suggests that UNC-40 has all of the signaling elements necessary to transduce the MADD-4 signal. Together, these observations suggest that EVA-1's role in mediating MADD-4 attraction must be UNC-40-dependent.

A model by which EVA-1 may counteract UNC-6's interference of the MADD-4-UNC-40 interaction emerges upon considering all of the data: EVA-1, UNC-40 and MADD-4 form a ternary complex that limits the ability of UNC-6 to engage UNC-40. It is unlikely that EVA-1 allosterically modulates UNC-40 to limit its interaction with UNC-6. If this were true, then removing EVA-1 should suppress some of the ventral muscle arm defects observed in MADD-4's absence because UNC-6 would then be free to engage UNC-40. However, suppression is not observed in the *eva-1 madd-4* double mutant, making it unlikely that EVA-1 antagonizes UNC-6's interaction with UNC-40 in a MADD-4-independent fashion. Furthermore, ectopically expressing EVA-1 in the distal tip cells in an otherwise wild type background failed to induce distal tip cell migration defects, providing further evidence that it is unlikely that EVA-1 reduces UNC-40's sensitivity to UNC-6 in any direct way. Instead, we infer that EVA-1 may limit UNC-6's access to UNC-40 by increasing the local concentration of MADD-4 and presenting it to UNC-40 within a ternary complex. This idea is consistent with our observation that in animals lacking UNC-6, EVA-1 facilitates MADD-4 function in muscles that are further away from the ventral nerve cord (and the source of endogenous MADD-4), but is superfluous in muscles that are closer to the ventral nerve cord.

EVA-1's role within the complex is unlikely to be limited to the presentation of MADD-4 to UNC-40 because an EVA-1 mutant protein lacking its cytoplasmic tail is non-functional, despite being able to bind UNC-40 and MADD-4. We speculate that EVA-1's cytoplasmic tail is needed to recruit or engage factors or processes that facilitate UNC-40-mediated signal transduction.

We have shown that EVA-1 functions with UNC-40 to direct muscle arm and AVM axon extensions towards the MADD-4 guidance cue. However, upon comparing lateral AVM axon guidance errors to dorsal AVM guidance errors in response to dorsally-expressed MADD-4, it is evident that EVA-1 plays two distinct roles in AVM axon guidance. Considering only the dorsal guidance of the AVM axon in response to dorsally-expressed MADD-4, EVA-1 functions with UNC-40 and independent of the SLT-1 pathway to mediate extension towards MADD-4. By contrast, when we consider only lateral AVM axon guidance errors, EVA-1 functions with SLT-1 in a pathway that is distinct from UNC-40. Together, these results suggest that EVA-1 can play simultaneous roles in distinct guidance pathways within the AVM neuron during axon extension.

EVA-1 was first identified as a co-receptor for SAX-3 (robo) within the SLT-1 (slit) pathway that helps guide the AVM and PVM axons ventrally [14]. EVA-1 co-immunoprecipitates with SAX-3 when co-expressed in cell culture, and SLT-1 interacts with both EVA-1 and SAX-3-expressing cells in culture [14]. We too found that SLT-1 bound SAX-3-expressing cells and that SLT-1 can bind EVA-1-expressing cells to a lesser degree. Compelling genetic analyses, which we replicated, indicate that EVA-1 functions within a SLT-1-SAX-3 pathway that operates in parallel to the UNC-6-UNC-40 pathway to guide AVM/PVM axons [14]. When combined, our data and that of Fujisawa et al demonstrates that EVA-1 functions within multiple guidance pathways and is capable of doing so in the same cell and possibly at the same time.

Why are multiple pathways needed to guide migrations along a single trajectory? One answer might be to provide increased precision. UNC-6 can be considered a global cue because ventral sources of UNC-6 can guide migrations along the entire circumference of the animal [9]. For example, UNC-6 guides circumferential motor axons from the ventral cord all the way to the dorsal cord [12]. By contrast, MADD-4's distribution must be more locally restricted than UNC-6 because ventral sources of MADD-4 do not interfere with dorsal muscle arm extension and vice versa [6]. Given that UNC-6 is a global cue and MADD-4 acts locally, the MADD-4-EVA-1-UNC-40 pathway may increase the precision by which cell processes reach their target. For example, as the ventral muscles extend arms towards the ventral cord within a field of UNC-6 ligand, EVA-1 may ensure that UNC-40 directs muscle arm extension to the source of MADD-4 expression at the ventral cord and is not confounded by the surrounding field of UNC-6 molecules. In dorsal muscles, EVA-1 might ensure that UNC-40 promotes muscle arm extension to the source of MADD-4 at the dorsal cord, and that UNC-40's effect is not diluted by ventrally-expressed UNC-6. In this way, migrating cells and cell extensions may capitalize upon multiple sources of spatial information while at the same time, guarding themselves against this information leading them astray.

## Materials and Methods

### Nematode Strains, Counts, and Microscopy

All strains were cultured and maintained at 20°C according to standard protocol [21]. All muscle arm counts were performed in the background of the chromosomally integrated transgenic array *trIs30*I or *trIs70*II, both of which express membrane-anchored YFP from the *him-4* promoter in select distal body wall muscles along with other markers of commissural motor axons [4]. See below for details on the *trIs* integrated transgenes. For transgenic strains, muscle arms were visualized either in the background of *trIs30*I or *trIs70*II, or directly from fluorescence associated with the expression of the transgenic array. To visualize YFP-tagged proteins, we used the Leica 513867 filter set; to visualize CFP-tagged proteins, we used the Leica 513866 filter set; to visualize RFPs, we used Leica's 513868 filter set. Muscle arms were counted as previously described [4]. AVMs and PVMs were visualized using the fluorescent reporter background *muIs32*II [22]. The direction of axon extension of 50 AVMs and PVMs from at least three separate populations of the same strain was counted from living animals on standard culture plates using an epifluorescent MZ16 dissection scope (Leica Inc.) with a 2× objective. Distal tip cell migration defects were scored by observing for the presence of anterior and posterior ventral white patches (relative to the vulva) from at least three separate populations of 50 living animals of the same strain. Distal tip cell migration defects were visualized using an epifluorescent MZ16 dissection scope (Leica Inc.) with a 2× objective. Redirected muscle arms were counted from 20 left and right sides of worms for each strain. All counts of muscle arms are from worms anaesthetized with 2–10 mM levamisole (Sigma) in M9 solution [23] and photographed as previously described [4]. During strain construction, genotypes like *madd-4(ok2862)* or *slt-1(ok255)*, which cannot be followed using obvious behavioral phenotypes, were instead followed using PCR and/or sequence of 5 or more individual progeny from a single cloned animal.

All statistical analyses were performed using a one-tailed, two-sample equal variance Student's t-test. We used a one-tailed test because for all analyses we hypothesized that the effect would be unidirectional. For example, when comparing muscle arm extension in mutants with wild type worms, we hypothesize that the mutant mean would be smaller than the wild type mean. Lastly, we chose a test that compared two independent samples of equal variance because all counts performed fall under normal distributions.

### A Forward Genetic Screen for Mutants that Suppress Muscle Arm Redirection towards CAN-Expressed MADD-4

A forward genetic screen for mutants that suppress muscle arm redirection towards CAN-expressed MADD-4 was performed by incubating a mixed stage population of RP1912 *madd-4(ok2854); trIs70*II; *trIs63*X animals in 50 µM ethyl methanesulfonate (EMS) for 4 hours. *trIs63* drives expression from the *ceh-23* promoter in the bisymmetrical pair of CAN neurons that extend axons along the lateral length of the worm. See below for details on the *trIs* integrated transgenes. Resulting F1s were synchronized as L1s [23] and grown on 6 cm plates (∼3000 per plate) for 3 days, and then transferred manually to each well of a 24-well plate. F2 progeny were screened 4–7 days later for mutants with defects in redirected muscle arm extensions using a Leica MZFLIII epiflourescence dissection microscope with a 2× objective. A total of ∼17,500 haploid genomes were screened clonally as described, and a further ∼2,400 haploid genomes were screened non-clonally by batch screening of F2 progeny. 51 suppressor mutants were isolated from these screens.

### Molecular Biology and Transgenics

The following chromosomally-integrated transgenic arrays were made using standard techniques [24], [25] and are available either from the *C. elegans* Genetic Centre or from us: *trIs30*[*pPRRF138.2(him-4p::Mb::YFP)*(10 ng/ul); *pPRZL44(hmr-1 bp::DsRed2)*(80 ng/ul); *pPR2.1(unc-129nsp::DsRed2)*(40 ng/ul)]I; *trIs34*[*pPRKC294(him-4p::UNC-40::YFP)*(2 ng/ul); *pRF4(rol-6(su1006))*(100 ng/ul)]X; *trIs41*[*pPRKC294(him-4p::UNC-40::YFP)*(125 ng/ul); *pRF4(rol-6(su1006))*(100 ng/ul)]II; *trIs63*[*pPRGS630(ceh-23p::MADD-4B::YFP)*(50 ng/ul); *pPRGS629(ceh-23p::YFP)*(50 ng/ul); *pPRGS382(myo-2p::mCherry)*(5 ng/ul)]X; *trIs65*[*pPRGS655(ceh-23p::MADD-4B::YFP)*(50 ng/ul); *pPRGS629(ceh-23p::YFP)*(50 ng/ul); *pPRGS382(myo-2p::mCherry)*(5 ng/ul)]; *trIs66*[*pPRGS628(unc-119p::MADD-4B::MYC::3XFLAG::YFP)*(50 ng/ul); *pPRGS382(myo-2p::mCherry)*(5 ng/ul)]X; *trIs69*[*pPRKC404(him-4p::PAT-2::CFP)*(100 ng/ul); *pRF4(rol-6(su1006))*(100 ng/ul)]; *trIs70*[*pPRZL138.2(him-4p::Mb::YFP)*(5 ng/ul); *pPR16(unc-25p::CFP)*(20 ng/ul); *pKS*(75 ng/ul)]II; *trIs73*[*pPRKC638.2(him-4p::UNC-40::MYC::3XFLAG::YFP)*(100 ng/ul)]; *trIs77*[*pPRGS698(unc-129msp::MADD-4A::YFP)*(12.5 ng/ul); *pPRGS699(unc-129msp::MADD-4B::YFP)*(12.5 ng/ul); *pPRGS382(myo-2p::mCherry)*(2 ng/ul)]; *trIs78*[*pPRGS698(unc-129msp::MADD-4A::YFP)*(12.5 ng/ul); *pPRGS699(unc-129msp::MADD-4B::YFP)*(12.5 ng/ul); *pPRGS382(myo-2p::mCherry)*(2 ng/ul)]IV; *trIs89*[*pPRKC793(him-4p::EVA-1::MYC::3XFLAG::CFP)*(50 ng/ul); *pPRGS382(myo-2p::mCherry)*(2 ng/ul); *pKS*(50 ng/ul)]. The constructs were built using standard protocols. Note that expression of MADD-4 and EVA-1 at relatively high concentrations can induce dominant muscle arm extension defects.

### Co-immunoprecipitation and Western Analysis

500 µL of mixed stage packed worms were washed thrice with M9 solution and once with PBS, and re-suspended in 1 mL ice cold solubilization buffer (25 mM Tris pH 7.4, 100 mM NaCl, 1 mM EDTA, 0.25% NP40, 1 mM PMSF, 1 mM Na3VO4, 2.5 µg/ml Pepstatin-A, 10 mM NaF and 1 protease inhibitor cocktail tablet (Roche) per 10 ml of solution). The samples were incubated on ice for 30 minutes, and lysed through three cycles of flash freezing in liquid nitrogen, partial thawing and sonnication for 5 seconds at 8 Watts. Next, the samples were incubated at 4°C with agitation for 30 minutes and then centrifuged (13000 rpm, 30 min, 4°C). A BCA assay (Thermo Scientific) was used to measure the protein concentration in the supernatant. 6 mg/ml of protein lysate was incubated with either 30 µl of packed Antiflag M2 agarose coupled antibody (Sigma) or 30 µl of packed Protein A/G agarose beads (SantaCruz) along with 2–4 µg of rabbit polyclonal anti-GFP antibody (GenScript) for 3 hrs with agitation at 4°C. The agarose beads were centrifuged and washed 5 times with 1 ml of cold solubilization buffer. Samples were resuspended in 2× Laemlli buffer and analyzed by western blot. The FLAG immunoprecipitated samples were analyzed using rabbit anti-GFP polyclonal antibodies (Genscript), and the GFP immunoprecipitated samples were analyzed using mouse anti-GFP monoclonal antibodies (SantaCruz).

Normalization of luciferase signal to protein level expression was performed by first subtracting control luciferase signal values from experimental luciferase signal values to obtain relative luciferase signal values. Next, protein level expression was measured by quantifying the number of pixels in a given protein band as visualized on film, using the publicly available software ImageJ (http://imagej.nih.gov/ij/). Normalized relative luciferase signal was obtained by expressing relative luciferase signal values as a fraction of protein level expression.

The integrated transgenes used in the co-immunoprecipitation experiments are as follows: *trIs41*[*pPRKC294(him-4p::UNC-40::YFP)*(125 ng/ul); *pRF4(rol-6(su1006))*(100 ng/ul)]II; *trIs69*[*pPRKC404(him-4p::PAT-2::CFP)*(100 ng/ul); *pRF4(rol-6(su1006))*(100 ng/ul)]; *trIs73*[*pPRKC638.2(him-4p::UNC-40::MYC::3XFLAG::YFP)*(100 ng/ul)]; *trIs89*[*pPRKC793(him-4p::EVA-1::MYC::3XFLAG::CFP)*(50 ng/ul); *pPRGS382(myo-2p::mCherry)*(2 ng/ul); *pKS*(50 ng/ul)].

The ability of EVA-1 truncations to co-immunoprecipitate UNC-40 was performed by establishing extrachromosomal transgenes in the *trIs41* integrated strain, and by following the aforementioned co-immunoprecipitation protocol: *pPRGS382(myo-2p::mCherry), pPRKC793(him-4p::EVA-1::MYC::3XFLAG::CFP), pPRJZ866(him-4p::EVA-1(GAL1+2)::MYC::3XFLAG::CFP), pPRJZ867(him-4p::EVA-1(CYTO)::MYC::3XFLAG::CFP), pPRJZ868(him-4p::EVA-1(GAL1)::MYC::3XFLAG::CFP), pPRJZ869(him-4p::EVA-1(GAL2)::MYC::3XFLAG::CFP), pPRAVS927.5(him-4p::EVA-1(PAT-2-TM)::MYC::3XFLAG::CFP)*. EVA-1 truncation constructs were all injected at 10 ng/ul together with *pPRGS382* at 2 ng/ul.

For protein level analysis of MADD-4 transgenes, total protein was obtained by sonnication and flash freezing worm lysates of respective integrated strains as described above. 20 ul containing 50 ng of total protein in 2× Laemmli sample buffer was analyzed by western blot. Blots were analyzed using rabbit anti-MADD-4 antibodies (which we made in conjunction with Genscript Inc. by injecting rabbits with MADD-4 peptides) and mouse anti-tubulin antibodies (Sigma) for the loading control.

### HEK293T Cell Surface Binding Experiments

The constructs for expressing the five receptors in HEK293T cells are as follows: *pPRGS861(CMV8-SS::3XFLAG::EVA-1); pPRGS831(CMV8-SS::3XFLAG::UNC-5(no death domain-* see [26]; *pPRGS757*(*pCDNA3-SS::3XFLAG::PAT-2*). *CMV3-SS:SAX-3::3XFLAG* and CMV3-SS::3XFLAG::UNC-40 were obtained from Joe Culotti [14]. The constructs for expressing the MADD-4B and SLT-1 from HEK293T cells are as follows: *pPRGS827(pCDNA3-SS::HA::MADD-4B::*Gaussia luciferase (Gluc)); and *pPRGS854(pCDNA3-SS::HA::SLT-1::*Gluc)). All expression plasmids have been sequence verified.

HEK293T cells were maintained and transfected in Dulbecco's Modified Eagle's Medium (Gibco) supplemented with 10% FBS (DMEM-10% FBS). All transfections were performed using JetPrime transfection reagent (Polyplus Transfection). Cell surface Gaussia luciferase binding assays were performed as previously reported [27].

To obtain concentrated conditioned culture media containing HA::MADD-4B::Gluc or Gluc::SLT-1::HA, 10 ug of respective expression plasmid was transfected into HEK293T cells, and culture media was changed to DMEM-0.2% FBS. After 24 hours of culture, the conditioned media was collected and centrifuged to remove cellular debris, and subsequently concentrated 10 times using Amicon Ultra-15 centrifugal filter units (Millipore). When necessary, ligands were immunoprecipitated from transfected cells by using an anti-HA affinity matrix (Roche) and analyzed by western blot using rabbit anti-HA antibodies (Sigma).

For the cell surface binding assays, 2 ug of individual plasmids expressing the various *C. elegans* receptors (previously described) were transfected into HEK293T cells. 48 hours post-transfection, culture media was removed and cells were incubated with concentrated conditioned media for 4 hours at 4°C. Cells were then harvested in a solution containing PBS, 0.2% BSA and protease inhibitors (Roche), and lysed in TNTE buffer (50 mM Tris, 150 mM NaCl, 1 mM EDTA, 10 mM NaF, 1 mM Na3VO4, 1 mM PMSF, 0.5% Triton X-100) containing protease inhibitors. *C. elegans* receptors were immunoprecipitated using mouse anti-FLAG antibodies (SIGMA), and were washed once in lysis buffer, and then in TNTE buffer containing 0.1% Triton X-100 plus protease inhibitor; five times for HA::MADD-4B::Gluc and 3 times for Gluc::SLT-1::HA ligands. One third of the immunoprecipitate was taken for Gaussia luciferase assays by using the BioLux Kit (NEB) and MicroLumat Plus LB96V luminometer (BERTHOLD). Remainder of the immunoprecipitates were analyzed by western blot using rabbit anti-Flag antibodies (NEB).

### MADD-4-EVA-1 Interaction Analysis *In Vivo*

To assay the ability of MADD-4::YFP (from the *trIs66* array) to localize to EVA-1::CFP expressing muscles (from the *trIs89* array), transgenic muscles were first identified by fluorescence under the CFP filter, and then re-examined under the YFP filter for presence of MADD-4::YFP. To assay the presence of MADD-4/EVA-1 vesicles, muscles were first identified for co-expression of both MADD-4::YFP (from the *trIs78* array) and EVA-1::CFP (from the *trIs89* array). These muscles were then examined for the presence of vesicles and were considered a positive score if containing more than two vesicles.

Colocalization studies between MADD-4/EVA-1 vesicles and endosomal markers were performed by creating strains that harbor *trIs89, trIs78* and either *huIs91* or *huIs97*. *huIs91* is a marker for early endosomes and *huIs97* is a marker for recycling endosomes; both transgenes were obtained from Rik Korswagen.

Integrated transgenes used in these colocalization studies are as follows: *trIs66*[*pPRGS628(unc-119p::MADD-4B::MYC::3XFLAG::YFP)*(50 ng/ul); *pPRGS382(myo-2p::mCherry)*(5 ng/ul)]X; *trIs78*[*pPRGS698(unc-129msp::MADD-4A::YFP)*(12.5 ng/ul); *pPRGS699(unc-129msp::MADD-4B::YFP)*(12.5 ng/ul); *pPRGS382(myo-2p::mCherry)*(2 ng/ul)]IV; *trIs89*[*pPRKC793(him-4p::EVA-1::MYC::3XFLAG::CFP)*(50 ng/ul); *pPRGS382(myo-2p::mCherry)*(2 ng/ul); *pKS*(50 ng/ul)]; *huIs91*[*myo-3p::mCherry::RAB-5*]; *huIs97*[*myo-3p::mCherry::RAB-11*]

### Test of EVA-1 Sufficiency in Guiding Distal Tip Cells towards MADD-4

To test sufficiency of EVA-1, we used the *emb-9* promoter similar to previous work [28] to drive EVA-1 expression in the distal tip cells from an extra-chromosomal array, which was first established in N2 wild type animals. Arrays were then crossed into RP2071 *unc-5(e53) trIs78IV; trIs30I* to generate *unc-5(e53) trIs78IV; trIs30I; trEx948 #1(or#2)[pPRKC948(emb-9p::EVA-1::CFP)(10 ng/ul); pPRGS365(myo-2p::CFP)(5 ng/ul)]* lines. Distal tip cell migration defects were scored as previously described only in transgenic animals. RNAi-by-feeding experiments were performed as previously reported [29].

### Characterizing Sub-cellular Localization of EVA-1 and UNC-40

Localization of EVA-1::CFP and UNC-40::YFP were examined under 63× magnification. Muscle expressed EVA-1::CFP was from an extrachromosomal array harboring *pPRGS394(him-4p::EVA-1::CFP)(10 ng/ul)* and *pPRGS382(myo-2p::mCherry)(2 ng/ul)*. Muscle expressed UNC-40::YFP came from the *trIs34* transgene.

## Supporting Information

Dataset S1 Contiguous dataset for figures presented throughout manuscript. Dataset S1 contains all relevant data with regards to Figures 1 through S4. Data for figures are separated into separate worksheets, and data points are given a number that corresponds with the raw data as presented in Dataset S2. Significance was determined using a two-sample equal variance Student's t-test.
(XLSX)

Dataset S2 Contiguous raw dataset for figures presented throughout manuscript. Dataset S2 contains all relevant raw data with regards to figures 1 through S4. Raw data for figures are separated into separate worksheets, and data points are indicated with a number that corresponds with that presented in Dataset S1. Significance was determined using a two-sample equal variance Student's t-test.
(XLSX)

Figure S1 Related to Figure 3b. Analyses of EVA-1 domain function. **A**. Quantification of VL11 muscle arm extension in young adults of the indicated genotype. Statistical significance (*p*<0.001) is indicated with a solid asterisk which is matched in colour with a dot above the data point to which the comparison was made. An outlined asterisk indicates a lack of significance. **B**. Analysis of the requirement of EVA-1's predicted galactose-binding lectin-like domains in binding luciferase-tagged MADD-4. The indicated version of FLAG-tagged EVA-1 was expressed in HEK293 cells, incubated with luc-tagged MADD-4-conditioned media, immunoprecipitated using antibodies against FLAG, and the resulting co-immunoprecipitated luciferase signal was measured (see the materials and methods section for more details). Statistical significance (*p*<0.01) is indicated with a solid asterisk which is matched in colour with a dot above the data point to which the comparison was made. An outlined asterisk indicates a lack of significance. **C**. Representative images of the ability of the indicated versions of muscle-expressed EVA-1::CFP to recruit neuronally-expressed (from the *trIs66* transgenic array) MADD-4::YFP to muscle cells. The orange arrows indicate MADD-4::YFP recruitment to EVA-1::CFP-expressing muscle cells. The red asterisks indicates gut auto-fluorescence. The scale bar represents 50 micrometers. **D**. A western blot showing an analysis of which EVA-1 domains are necessary to maintain an interaction with UNC-40 as determined through co-immunoprecipitation of muscle-expressed proteins. All versions of EVA-1 shown are able to co-immunoprecipitate UNC-40. Only the substitution of EVA-1's transmembrane domain for that of PAT-2 abolishes EVA-1's interaction with UNC-40 (see Figure 4b).
(TIF)

Figure S2 Related to Figure 3e–3m. MADD-4 induces EVA-1 endocytosis in a dose-dependent manner. **A**. A western blot of lysates of five strains harbouring the indicated transgenic arrays probed and with anti-MADD-4 antibodies. The MADD-4A and MADD-4B isoforms are indicated with arrowheads. β-tubulin is used as a loading control. Only *trIs66* (neuronally-expressed MADD-4B) and *trIs78* (MADD-4A and MADD-4B expressed from the dorsal muscles) are of relevance here. A quantification of the relative abundance of the proteins (normalized to tubulin) is shown above the blot. **B**. A strain expressing muscle specific MADD-4::YFP (from the *trIs78* array), EVA-1::CFP (from the *trIs89* array) and mCherry::RAB-11 (from the *huIs97* array). Arrows indicate vesicles in which all three markers co-localize. **C**. A strain expressing muscle specific MADD-4::YFP (from the *trIs78* array), EVA-1::CFP (from the *trIs89* array) and mCherry::RAB-5 (from the *huIs91* array). Arrows indicate randomly chosen vesicles in which all three markers co-localize. The scale bar represents 50 µM.
(TIF)

Figure S3 Related to Figure 4. Characterizing the sub-cellular localization and interaction specificity of EVA-1 and UNC-40. **A**. Muscle-expressed EVA-1::CFP (from an extrachromosomal array) remains localized to muscle arm termini (arrows) in the *unc-40(n324)* null mutant. **B**. Muscle-expressed UNC-40::YFP (from the *trIs34* transgene) remains localized to muscle arm termini (arrows) in the *eva-1(tr301)* null mutant. **C**. A western blot showing that EVA-1 will not co-immunoprecipitate with PAT-2. **D**. A western blot showing that UNC-40 will not co-immunoprecipitate with PAT-2. The scale bar represents 50 µM.
(TIF)

Figure S4 Related to Figure 5. EVA-1 is sufficient to confer UNC-40 sensitivity to MADD-4 in the background of UNC-6. Anterior distal tip cell migration defects either in the presence (+) or absence (−) of dorsal muscle-expressed (m)MADD-4 (from the *trIs78* transgenic array) for the indicated genotype. The posterior distal defects are shown in Figure 5e and for simplicity of display, the anterior defects are reported here. The purpose of the ‘data point’ row is to provide clarity within main text. EVA-1::CFP was expressed in the distal tip cells from the *emb-9* promoter as was previously done for UNC-5 [28]. The two extrachromosomal arrays are called *trEx948#1* ([DTC-EVA-1]#1 on the graph) and *trEx948#2* ([DTC-EVA-1]#2 on the graph). [28]. For each graph, statistical significance (*p*<0.05) is documented as described for figure 1f. Standard error of the mean is shown in both graphs.
(TIF)
