# Supplementary figures and images for: EVA-1 Functions as an UNC-40 Co-receptor to Enhance Attraction to the MADD-4 Guidance Cue in Caenorhabditis elegans
Source: PLoS Genet. 2014 Aug 14;10(8):e1004521. doi: 10.1371/journal.pgen.1004521 (PMC4133157; doi:10.1371/journal.pgen.1004521)

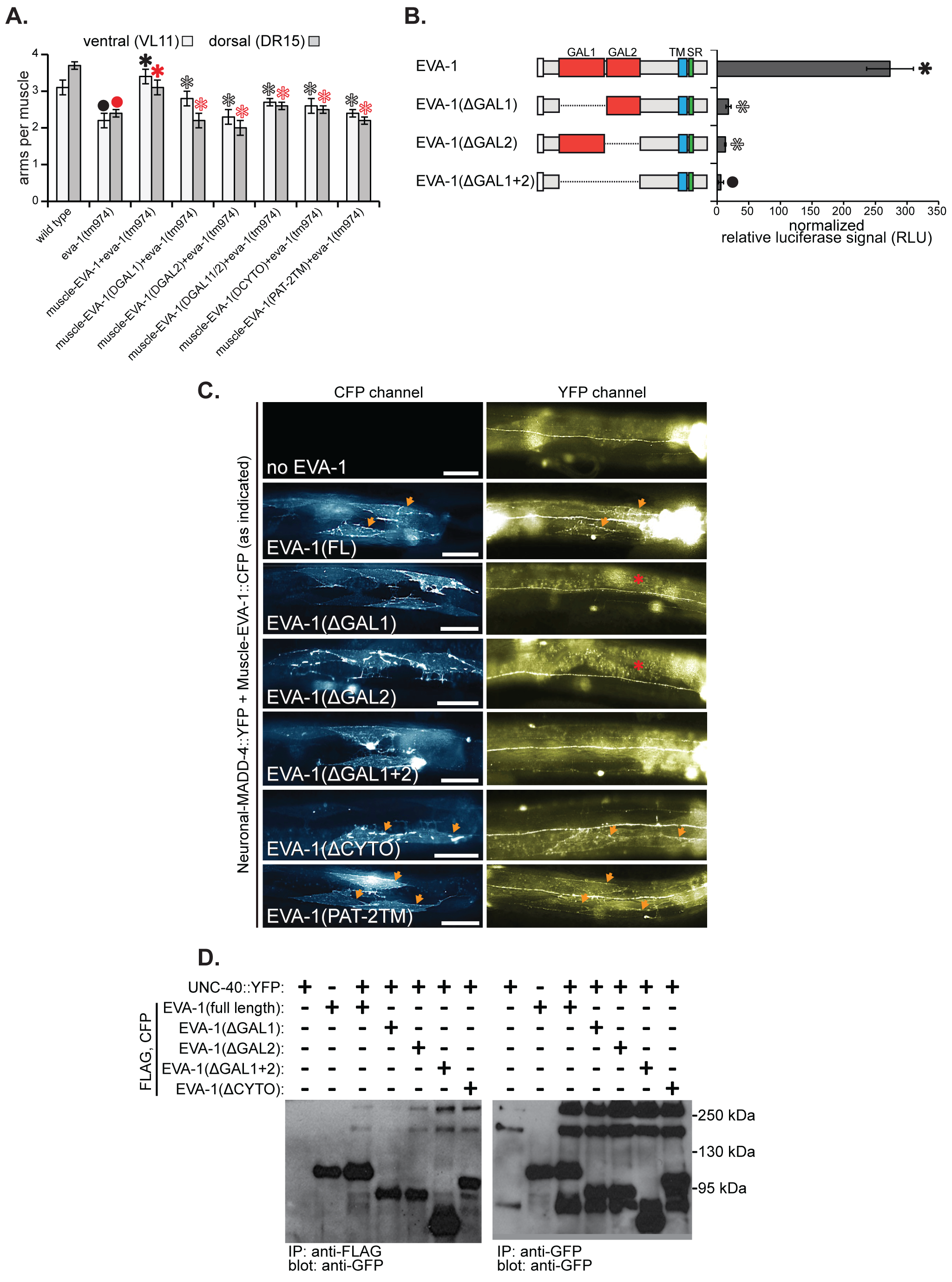

Supplement: Figure S1 — Related to Figure 3b. Analyses of EVA-1 domain function. A. Quantification of VL11 muscle arm extension in young adults of the indicated genotype. Statistical significance (p<0.001) is indicated with a solid asterisk which is matched in colour with a dot above the data point to which the comparison was made. An outlined asterisk indicates a lack of significance. B. Analysis of the requirement of EVA-1's predicted galactose-binding lectin-like domains in binding luciferase-tagged MADD-4. The indicated version of FLAG-tagged EVA-1 was expressed in HEK293 cells, incubated with luc-tagged MADD-4-conditioned media, immunoprecipitated using antibodies against FLAG, and the resulting co-immunoprecipitated luciferase signal was measured (see the materials and methods section for more details). Statistical significance (p<0.01) is indicated with a solid asterisk which is matched in colour with a dot above the data point to which the comparison was made. An outlined asterisk indicates a lack of significance. C. Representative images of the ability of the indicated versions of muscle-expressed EVA-1::CFP to recruit neuronally-expressed (from the trIs66 transgenic array) MADD-4::YFP to muscle cells. The orange arrows indicate MADD-4::YFP recruitment to EVA-1::CFP-expressing muscle cells. The red asterisks indicates gut auto-fluorescence. The scale bar represents 50 micrometers. D. A western blot showing an analysis of which EVA-1 domains are necessary to maintain an interaction with UNC-40 as determined through co-immunoprecipitation of muscle-expressed proteins. All versions of EVA-1 shown are able to co-immunoprecipitate UNC-40. Only the substitution of EVA-1's transmembrane domain for that of PAT-2 abolishes EVA-1's interaction with UNC-40 (see Figure 4b). (TIF) [file pgen.1004521.s003.tif]

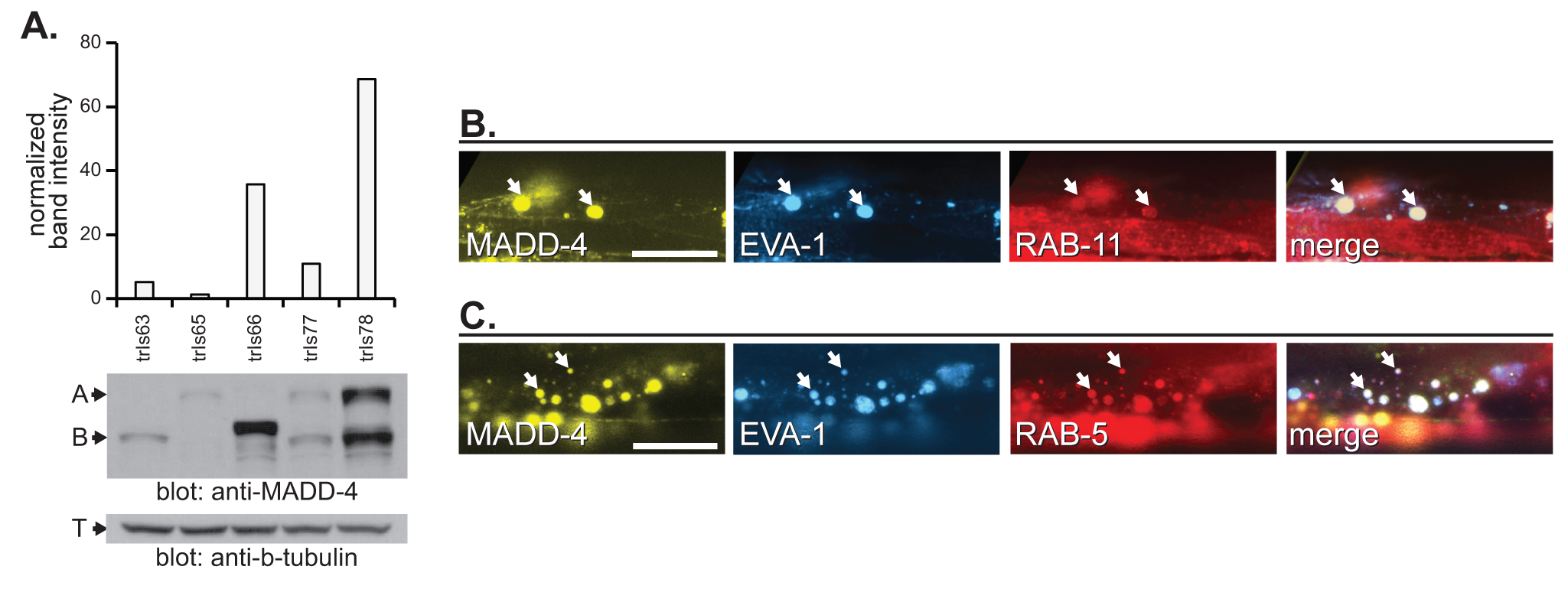

Supplement: Figure S2 — Related to Figure 3e–3m. MADD-4 induces EVA-1 endocytosis in a dose-dependent manner. A. A western blot of lysates of five strains harbouring the indicated transgenic arrays probed and with anti-MADD-4 antibodies. The MADD-4A and MADD-4B isoforms are indicated with arrowheads. β-tubulin is used as a loading control. Only trIs66 (neuronally-expressed MADD-4B) and trIs78 (MADD-4A and MADD-4B expressed from the dorsal muscles) are of relevance here. A quantification of the relative abundance of the proteins (normalized to tubulin) is shown above the blot. B. A strain expressing muscle specific MADD-4::YFP (from the trIs78 array), EVA-1::CFP (from the trIs89 array) and mCherry::RAB-11 (from the huIs97 array). Arrows indicate vesicles in which all three markers co-localize. C. A strain expressing muscle specific MADD-4::YFP (from the trIs78 array), EVA-1::CFP (from the trIs89 array) and mCherry::RAB-5 (from the huIs91 array). Arrows indicate randomly chosen vesicles in which all three markers co-localize. The scale bar represents 50 µM. (TIF) [file pgen.1004521.s004.tif]

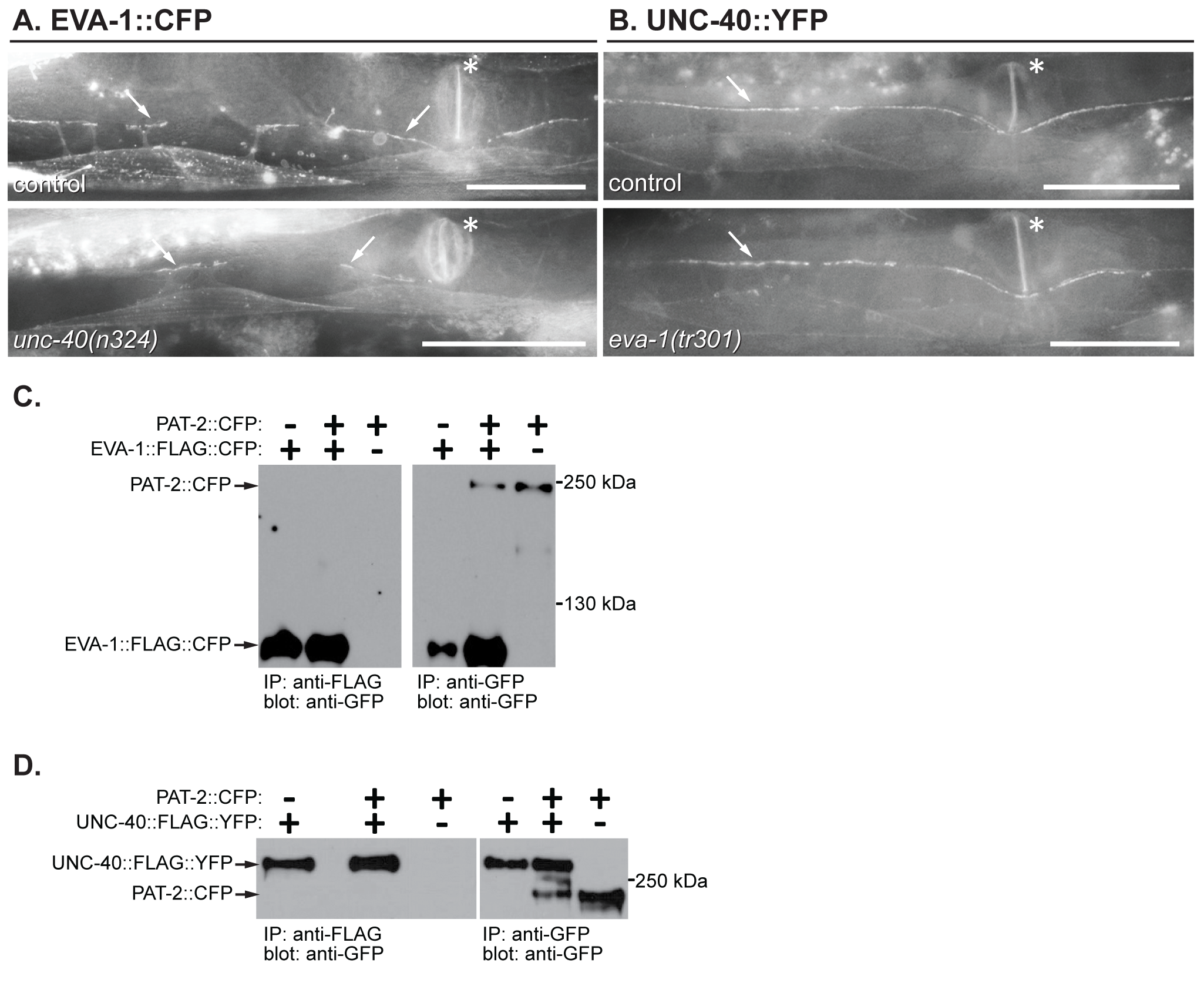

Supplement: Figure S3 — Related to Figure 4. Characterizing the sub-cellular localization and interaction specificity of EVA-1 and UNC-40. A. Muscle-expressed EVA-1::CFP (from an extrachromosomal array) remains localized to muscle arm termini (arrows) in the unc-40(n324) null mutant. B. Muscle-expressed UNC-40::YFP (from the trIs34 transgene) remains localized to muscle arm termini (arrows) in the eva-1(tr301) null mutant. C. A western blot showing that EVA-1 will not co-immunoprecipitate with PAT-2. D. A western blot showing that UNC-40 will not co-immunoprecipitate with PAT-2. The scale bar represents 50 µM. (TIF) [file pgen.1004521.s005.tif]

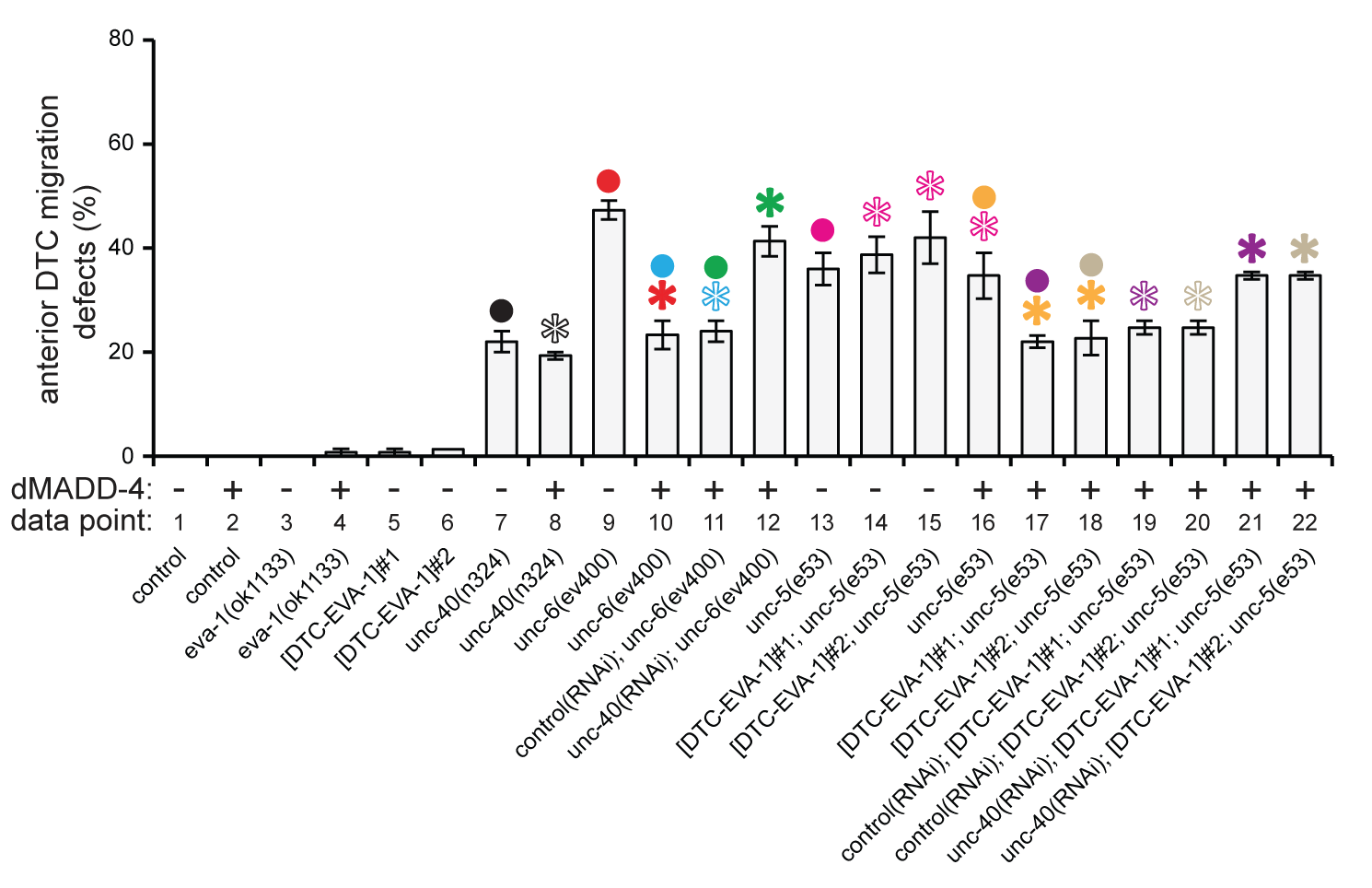

Supplement: Figure S4 — Related to Figure 5. EVA-1 is sufficient to confer UNC-40 sensitivity to MADD-4 in the background of UNC-6. Anterior distal tip cell migration defects either in the presence (+) or absence (−) of dorsal muscle-expressed (m)MADD-4 (from the trIs78 transgenic array) for the indicated genotype. The posterior distal defects are shown in Figure 5e and for simplicity of display, the anterior defects are reported here. The purpose of the ‘data point’ row is to provide clarity within main text. EVA-1::CFP was expressed in the distal tip cells from the emb-9 promoter as was previously done for UNC-5 [28]. The two extrachromosomal arrays are called trEx948#1 ([DTC-EVA-1]#1 on the graph) and trEx948#2 ([DTC-EVA-1]#2 on the graph). [28]. For each graph, statistical significance (p<0.05) is documented as described for figure 1f. Standard error of the mean is shown in both graphs. (TIF) [file pgen.1004521.s006.tif]
